# Electronic Medical Record Cancer Incidence over Six Years Comparing New Users of Glargine with New Users of NPH Insulin

**DOI:** 10.1371/journal.pone.0109433

**Published:** 2014-10-20

**Authors:** Soo Lim, Katherine G. Stember, Wei He, Porneala C. Bianca, Carine Yelibi, Alison Marquis, Til Stürmer, John B. Buse, James B. Meigs

**Affiliations:** 1 General Medicine Division, Department of Medicine, Massachusetts General Hospital and Harvard Medical School, Boston, Massachusetts, United States of America; 2 Department of Internal Medicine, Seoul National University College of Medicine and Seoul National University Bundang Hospital, Seongnam, Korea; 3 Center for Human Genetic Research, Department of Medicine, Massachusetts General Hospital and Harvard Medical School, Boston, Massachusetts, United States of America; 4 Division of Endocrinology, University of North Carolina School of Medicine, Chapel Hill, North Carolina, United States of America; 5 Department of Epidemiology, Gillings School of Global Public Health, University of North Carolina, Chapel Hill, North Carolina, United States of America; University of Algarve, Portugal

## Abstract

**Background:**

Recent studies suggested that insulin glargine use could be associated with increased risk of cancer. We compared the incidence of cancer in new users of glargine versus new users of NPH in a longitudinal clinical cohort with diabetes for up to 6 years.

**Methods and Findings:**

From all patients who had been regularly followed at Massachusetts General Hospital from 1/01/2005 to 12/31/2010, 3,680 patients who had a medication record for glargine or NPH usage were obtained from the electronic medical record (EMR). From those we selected 539 new glargine users (age: 60.1±13.6 years, BMI: 32.7±7.5 kg/m^2^) and 343 new NPH users (61.5±14.1 years, 32.7±8.3 kg/m^2^) who had no prevalent cancer during 19 months prior to glargine or NPH initiation. All incident cancer cases were ascertained from the EMR requiring at least 2 ICD-9 codes within a 2 month period. Insulin exposure time and cumulative dose were validated. The statistical analysis compared the rates of cancer in new glargine vs. new NPH users while on treatment, adjusted for the propensity to receive one or the other insulin. There were 26 and 28 new cancer cases in new glargine and new NPH users for 1559 and 1126 person-years follow-up, respectively. There were no differences in the propensity-adjusted clinical characteristics between groups. The adjusted hazard ratio for the cancer incidence comparing glargine vs. NPH use was 0.65 (95% CI: 0.36–1.19).

**Conclusions:**

Insulin glargine is not associated with development of cancers when compared with NPH in this longitudinal and carefully retrieved EMR data.

## Introduction

Four studies investigating the association of glargine insulin treatment with cancers, which were published in the journal *Diabetologia* in 2009, created concern for both physicians and patients with diabetes [1]–[4]. Since the publication of these studies, many subsequent studies, meta-analyses, and editorials have followed [5]–[12]. One study looking at the effects of glargine vs. neutral protamine Hagedorn (NPH) on the progression of diabetic retinopathy showed similar cancer incidence during 5 years between the two treatment groups [9]. Another study based on the French National Healthcare Insurance system database also showed no excess risk of cancer in patients with type 2 diabetes on insulin glargine compared with those on human insulin [8]. This study reported that the overall risk of cancer in patients treated with insulin glargine was about half that of patients with human insulin. Interestingly, another study from the Netherlands found that insulin glargine use was associated with a 25% lower risk of malignancies when compared with human insulin while a 58% increased risk in insulin glargine group was found for breast cancer [7]. The Outcome Reduction with an Initial Glargine Intervention (ORIGIN) trial showed exactly the same number of cancer cases with glargine treatment vs. standard care without insulin treatment over 6 years in subjects with prediabetes or early diabetes [13]. More recently, data from French national health insurance databases indicate that in a large cohort of more than 70,000 patients newly treated by long-acting insulin, there was no increased risk of cancer observed in insulin glargine users compared with other long-acting insulin users [14]. Another recent study with more than 40,000 patients covered by the Inovalon Medical Outcomes Research for Effectiveness and Economics Registry showed that patients initiating insulin glargine treatment did not show increased risk of cancer compared with NPH users [15]. A caveat to these studies, however, is that they are limited by relatively short follow-up work and consequently lack of an accurate reflection of insulin prescription patterns.

In this study we reinvestigated the hypothesis that insulin glargine has an impact on cancer incidence in patients with diabetes via a new and improved approach that utilizes patient electronic medical record (EMR) data. This approach was designed to elucidate this relationship by improving on the limitations of the previously mentioned conflicting studies, including poorly defined drug and dose information, short study duration, and a lack of control of potential confounding factors.

## Subjects and Methods

### 2.1. Study population

The study was approved by the Institutional Review Boards of the University of North Carolina at Chapel Hill and of the Partners Healthcare System-MGH. The patient records/information was anonymized and de-identified prior to analysis. Massachusetts General Hospital (MGH) utilizes an EMR system that allows for storage, retrieval, and modification of patient health records. The EMR contains a plethora of health information for patients including anthropometric parameters, lifestyle features, diseases, laboratory results, and details of prescription of medication. From this database, we retrieved data for 20,087 patients with diabetes aged at least 18 years old, and who were loyal to MGH at 2005 and thereafter.

Patients were classified as loyal if they were connected either to a specific attending primary care physician or to the practice where they receive the majority of their care [16]. Loyal patients with diabetes were defined as patients with diabetes who had at least 2 consecutive years of loyal status. Among these loyal patients with diabetes, 3,680 had at least one medication record for glargine or NPH after 1/1/2005 and before 12/31/2010.

Of these patients, 2,285 had data from three 6-month periods prior to the first record for glargine or NPH. In order to be considered a new user, patients were required to have a 19 month time period prior to glargine/NPH initiation where they did not take any insulin (except a maximum of one prescription for short-acting insulin). The 19-month time period was determined by allowing for a one-month supply from the patient’s previous insulin treatment, a six-month grace period, and the one-year wash-out period. After excluding patients with an insulin prescription during the 19 months prior to glargine or NPH initiation and patients with any evidence of prevalent cancer during this period, we found 578 new glargine users and 415 new NPH users. From these patients, we selected 539 (glargine) and 343 (NPH) patients who had HbA1c levels at the time of enrollment. This eligibility cohort algorithm is shown in **Figure A in File S1**.

### 2.2. Measurement of outcomes

The primary outcome in this study was the development of any cancer excluding non-melanoma skin cancer. We excluded cases of non-melanoma skin cancer in the analysis as it is not likely to be influenced by insulin treatment biologically [14], [15]. The only carcinoma *in situ* (CIS) that were included in the primary outcome were cases of CIS breast cancer (a complete list of ICD-9 cancer codes is included in **Table A in File S1**).

To identify incident cancer cases after initiation of glargine or NPH insulin, we abstracted ICD-9 cancer codes from the EMR. To ensure that patients were correctly allotted into either the cancer or no cancer group, we used modified criteria as proposed by Setoguchi et al., which require at least 2 ICD-9 cancer codes on two different dates within a 2 month period to diagnose cancer [17]. Requiring two codes significantly reduces the number of cases that are identified in error (false positives). As a validation of the use of the Setoguchi et al. criteria to identify cancer cases in our database, we randomly selected 120 patients with diabetes who were diagnosed with one of 5 cancers (breast, prostate, colon, pancreas, bladder, and kidney cancers) using the modified Setoguchi criteria, and 20 patients with diabetes but without cancer. We then manually assessed the EMR for each patient to determine their cancer status. If patients did in fact have a specific cancer, a diagnosis date was recorded. In these two groups of patients with and without cancer, we found 99% sensitivity and 95% specificity in the diagnosis of cancer using the Setoguchi criteria as compared to our manually assessed records.

### 2.3. Measurement of exposure

#### Insulin use and dose

From the EMR database we collected data for glargine and NPH use that included dose as well as start and end date of prescription. Parallel use of other insulin was also investigated. The effect of cumulative insulin dose was addressed by calculating the cumulative insulin dose as of every new insulin record for glargine or NPH and categorizing patients into mutually exclusive categories of cumulative dose (e.g., 0−<10 kU, 10−<20 kU, 20−<50 kU, ≥50 kU). Cumulative dose was based exclusively on the medication records for either glargine or NPH (whichever long-acting insulin was initiated); dose of short-acting insulin was not included. Cumulative dose was calculated until a patient stopped taking long-acting insulin, augmented with different long-acting insulin, switched to other long-acting insulin or the study ended (whichever came first). We also gathered other diabetes medication information including name of drug and dose, and date that it was prescribed from the EMR data.

#### Validation of all other major clinical exposures

Anthropometric and biochemical parameters such as body mass index (BMI), blood pressures, serum hemoglobin, liver enzyme activities, serum creatinine, fasting glucose, glycosylated hemoglobin (HbA1c), and lipid profiles were measured during usual clinical care. All covariates such as duration of diabetes, smoking status, comorbidity, and medication were defined based on data from the one year period prior to the start date of NPH or glargine. The only exception was BMI; in this case, the latest available value prior to or on the start date of NPH or glargine was used when available. In 3.4% of patients, the earliest BMI value within the two years after the start of NPH or glargine was used. Medical information including frequency of clinic visit, number of hospitalizations for any reason, and total number of days in the hospital for any reason, was also obtained from the EMR database.

We used Natural Language Processing (NLP) to gather smoking information from the discharge summaries in the EMR [18]. Only NLP records prior to the start date of NPH or glargine were used in order to assess the smoking status of a new user before the start date of diabetes treatment. We summarized the data so that each patient had a smoking status for each calendar year. We considered a patient ‘current smoker’ if he or she had a current smoking status in the glargine or NPH start date year. Patients with a current smoker status in the years prior to the start date of glargine or NPH were considered ‘past smokers’.

### 2.4. Statistical analysis

First, we compared clinical characteristics including age, sex, duration of diabetes, BMI, smoking status, medications, screening tests for cancer, and the number of physician visit between new glargine users and new NPH users (**Table 1**). To adjust for potential confounding due to channeling between the insulin glargine and NPH, the propensity score for glargine initiation was estimated for all variables using logistic regression models [19]. Briefly, the stabilized inverse probability of treatment weights was calculated. We then applied the weights and checked that the treatment groups were balanced in terms of the covariates in the weighted pseudo-population (**Table 1**).

**Table 1:** Baseline unadjusted and propensity score weighted characteristics of new glargine and NPH insulin users.

	Unadjusted Values		Propensity Score Weighted Values	
Demographic Characteristic	Glargine N = 539	NPH N = 343	Glargine	NPH
Age, mean (SD)	60.1 (13.6)	61.5 (14.1)	60.3 (14.0)	59.9 (14.8)
Duration of diabetes (years), mean (SD)	8.2 (5.7)	8.2 (5.8)	8.3 (5.6)	8.1 (5.8)
Baseline BMI (kg/m^2^), mean (SD)	32.7 (7.5)	32.7 (8.3)	32.5 (7.6)	32.2 (8.1)
HbA1c category, n (%)				
≤7.5% or 58 mmol/mol	103 (19.1)	88 (26.0)	142 (26.1)	88 (26.0)
7.5% or 58 mmol/mol<∼≤8.6% or 70 mmol/mol	158 (29.3)	89 (26.4)	150 (27.6)	89 (26.4)
8.6% or 70 mmol/mol<∼≤10.2% or 88 mmol/mol	173 (32.1)	90 (26.7)	145 (26.7)	90 (26.7)
>10.2% or 88 mmol/mol	105 (19.5)	70 (20.9)	106 (19.6)	70 (20.9)
Women, n (%)	261 (48.4)	165 (49.1)	267 (49.2)	165 (49.1)
White, n (%)	382 (70.9)	257 (74.9)	393 (72.3)	246 (73.0)
Black, n (%)	36 (6.7)	28 (8.2)	38 (7.0)	22 (6.5)
Smoking category, n (%)				
Never smoker	205 (38.0)	112 (32.7)	192 (35.4)	128 (38.1)
Past smoker	163 (30.2)	128 (37.3)	183 (33.6)	106 (31.3)
Current smoker	171 (31.7)	103 (30.0)	169 (31.0)	103 (30.6)
Year of cohort entry, n (%)				
2005	47 (8.7)	58 (16.9)	68 (12.5)	42 (12.6)
2006	87 (16.1)	80 (23.3)	99 (18.3)	68 (20.2)
2007	123 (22.8)	87 (25.4)	132 (24.4)	76 (22.5)
2008	161 (29.9)	66 (19.2)	138 (25.4)	90 (26.7)
2009	121 (22.4)	52 (15.2)	106 (19.5)	61 (18.1)
***Comorbidity***				
Pulmonary disease, n (%)	73 (13.5)	75 (21.9)	89 (16.3)	55 (16.3)
Congestive heart failure, n (%)	62 (11.5)	68 (19.8)	82 (15.1)	51 (15.2)
Diabetic retinopathy, n (%)	20 (3.7)	20 (5.8)	24 (4.4)	16 (4.6)
Diabetic nephropathy, n (%)	76 (14.1)	69 (20.1)	96 (17.7)	56 (16.6)
Diabetic neuropathy, n (%)	45 (8.3)	60 (17.5)	64 (11.8)	41 (12.1)
***Medication***				
Metformin, n (%)	385 (71.4)	192 (56.0)	360 (66.3)	224 (66.6)
Thiazolidinediones, n (%)	159 (29.5)	59 (17.2)	128 (23.6)	73 (21.7)
Sulfonylureas, n (%)	385 (71.4)	169 (49.3)	337 (62.0)	199 (59.2)
Other hypoglycemic agents, n (%)	40 (7.4)	17 (5.0)	33 (6.0)	15 (4.4)
Statins, n (%)	393 (72.9)	247 (72.0)	387 (71.3)	234 (69.4)
Bile acid medications, n (%)	8 (1.5)	3 (0.9)	6 (1.1)	3 (0.8)
Fibrates, n (%)	66 (12.2)	33 (9.6)	61 (11.2)	32 (9.5)
Niacin, n (%)	11 (2.0)	6 (1.7)	14 (2.6)	9 (2.7)
Other lipid-lowering medications, n (%)	40 (7.4)	18 (5.2)	39 (7.2)	22 (6.6)
Testosterone, n (%)	5 (0.9)	1 (0.3)	3 (0.6)	1 (0.3)
Estrogen, n (%)	11 (2.0)	4 (1.2)	8 (1.6)	3 (1.0)
Estradiol, n (%)	13 (2.4)	8 (2.3)	12 (2.2)	6 (1.8)
Oral contraceptives, n (%)	16 (3.0)	11 (3.2)	16 (2.9)	8 (2.4)
Progesterone/Progestin, n (%)	13 (2.4)	12 (3.5)	14 (2.5)	8 (2.3)
Cardiac glycosides, n (%)	27 (5.0)	28 (8.2)	31 (5.8)	19 (5.7)
ACE inhibitors/ARBs, n (%)	410 (76.1)	256 (74.6)	406 (74.6)	245 (72.8)
Diuretics (loop), n (%)	105 (19.5)	102 (29.7)	129 (23.8)	77 (22.8)
Diuretics (non-loop), n (%)	211 (39.1)	175 (51.0)	236 (43.5)	143 (42.4)
Anticholinergics, n (%)	41 (7.6)	43 (12.5)	50 (9.2)	32 (9.6)
Beta 2 agonist, n (%)	125 (23.2)	96 (28.0)	132 (24.3)	80 (23.7)
Theophylline, n (%)	14 (2.6)	13 (3.8)	18 (3.3)	11 (3.2)
Corticosteroid, n (%)	113 (21.0)	103 (30.0)	132 (24.2)	82 (24.3)
Anti-depressants, n (%)	219 (40.6)	130 (37.9)	220 (40.6)	135 (40.0)
Beta blockers, n (%)	267 (49.5)	177 (51.6)	276 (50.8)	164 (48.8)
Calcium channel blockers, n (%)	126 (23.4)	113 (32.9)	145 (26.8)	91 (27.2)
***Medical history***				
Number of Patients with each of the Following Tests, n (%)				
Colonoscopy	59 (10.9)	33 (9.6)	59 (10.8)	39 (11.6)
Pap Smear	76 (14.1)	53 (15.5)	84 (15.5)	51 (15.2)
PSA Test	124 (23.0)	70 (20.4)	124 (22.9)	79 (23.6)
Mammogram	132 (24.5)	76 (22.2)	127 (23.5)	71 (21.1)
ECG	268 (49.7)	208 (60.6)	297 (54.6)	184 (54.7)
Cholesterol Test	490 (90.9)	313 (91.3)	495 (91.2)	297 (88.3)
Flu vaccine	276 (51.2)	163 (47.5)	266 (49.0)	177 (52.6)
Fecal occult blood test	69 (12.8)	48 (14.0)	74 (13.7)	45 (13.3)
Primary care physician visits, n (%)				
0	167 (31.0)	118 (34.4)	174 (32.0)	103 (30.6)
1–3	95 (17.6)	84 (24.5)	115 (21.2)	71 (21.1)
4–6	167 (31.0)	82 (23.9)	147 (27.1)	91 (27.0)
>6	110 (20.4)	59 (17.2)	107 (19.7)	72 (21.4)
Diabetes center physician visits, n (%)				
0	485 (90.0)	253 (73.8)	454 (83.7)	280 (83.0)
1–3	34 (6.3)	52 (15.2)	54 (10.0)	35 (10.5)
4–6	15 (2.8)	30 (8.7)	25 (4.7)	17 (5.0)
>6	5 (0.9)	8 (2.3)	9 (1.7)	5 (1.4)

Note: Only patients with nonmissing values for all covariates are included. All variables were included in the logistic regression for propensity score.

We used an intent-to-treat approach for the primary analysis of the relationship between insulin use and cancer incidence. We tested incidence rates of cancers in new glargine users versus new NPH users from the time of drug initiation until the time point of: (1) diagnosis of any cancer (except non-melanoma skin cancer), (2) death, (3) termination of health care at MGH (defined as one year past the last available visit date in the database), or (4) the end of the study (December 31, 2010); whichever came first. The association between insulin use and cancer incidence was analyzed in the weighted pseudo-population using Cox proportional hazard models.

For further analysis, we included cumulative dose as a time-varying covariate in the Cox proportional hazards model. With every new record for glargine or NPH (whichever treatment was initiated), cumulative dose was calculated and patients were categorized into mutually exclusive categories of cumulative dose (0−10 kU, ≥10−20 kU, ≥20−50 kU, ≥50 kU etc). Time at risk for incident cancer began once a patient reached the corresponding cumulative dose and ended once a patient reached the next cumulative dose level. If a patient had a cancer event, it was counted within the dose category when it occurred. All data were analyzed using SAS Version 9.2 (SAS, Cary, NC, USA). The nominal level of significance for all analyses was P<0.05.

## Results

Table 1 shows baseline characteristics of new glargine and new NPH users. After propensity score implementation, all demographic variables were balanced across treatment cohorts. The current smoker at the baseline was about one third in both groups. Co-morbid conditions including diabetic complications were also not statistically different between two groups after propensity score implementation. Similarly, oral antidiabetic medications, particularly metformin, were comparable between glargine users and NPH users in the prior medication usage. There was no difference in the number of patients who received cancer screening tests in either crude or propensity score implemented comparisons. In comparison of health care utilization 1 year prior to index prescription, the total number of primary care center visits and diabetes center visits were not different between glargine users and NPH users (**Table 1**). Thus, after adjustment for the propensity to have been treated with glargine versus NPH, there was no difference in rates or proportions of important clinical characteristics related with cancers comparing new glargine users with new NPH users.

The range of follow-up was 1–71 months and the median follow-up was 37.2 months (34.7 and 39.4 months in glargine and NPH group, respectively). Twenty three patients died during follow-up. As primary outcome, we found 54 incident cancer cases occurring after the new use of insulin in intent-to-treat analysis (**Table 2**). Of these cases, 26 events occurred in the glargine group and 28 occurred in the NPH group. Breast and prostate cancers were most common and colon, pancreas, lung, and kidney cancers followed next (**Table A in File S1**). After accounting for potential clinical confounders, the hazard ratio for incidence cancer comparing new glargine with new NPH users was 0.65 (95% confidence interval 0.36, 1.19), indicating no increased risk of cancer in new glargine users compared with new NPH users.

**Table 2:** Comparison of cancer incidence between new glargine and NPH users (Intent-to-Treat Analysis).

Endpoint	Treatment	MeanFollow-upTime (years)	Events	TotalPerson-years	Incidence ratefor 100person-years	UnadjustedHR (95% CI)	AdjustedHR (95% CI)
Any cancer	Glargine	2.89	26	1559	1.67	0.67	0.65
	(n = 539)					(0.39, 1.14)	(0.36, 1.19)
	NPH	3.28	28	1126	2.49		
	(n = 343)						

CI, confidence interval; HR, hazard ratio. Note: Unadjusted HR and 95% CI were obtained from a Cox proportional hazards model adjusting for treatment only. Adjusted HR and 95% CI were obtained from a Cox proportional hazards model that adjusted for all variables listed in table 1 using inverse probability of treatment weights. Patients were followed until the first occurrence of one of the following events: the patient developed cancer, death, the study ended, or one year after the patient’s last visit in the database.

**Figure 1:**
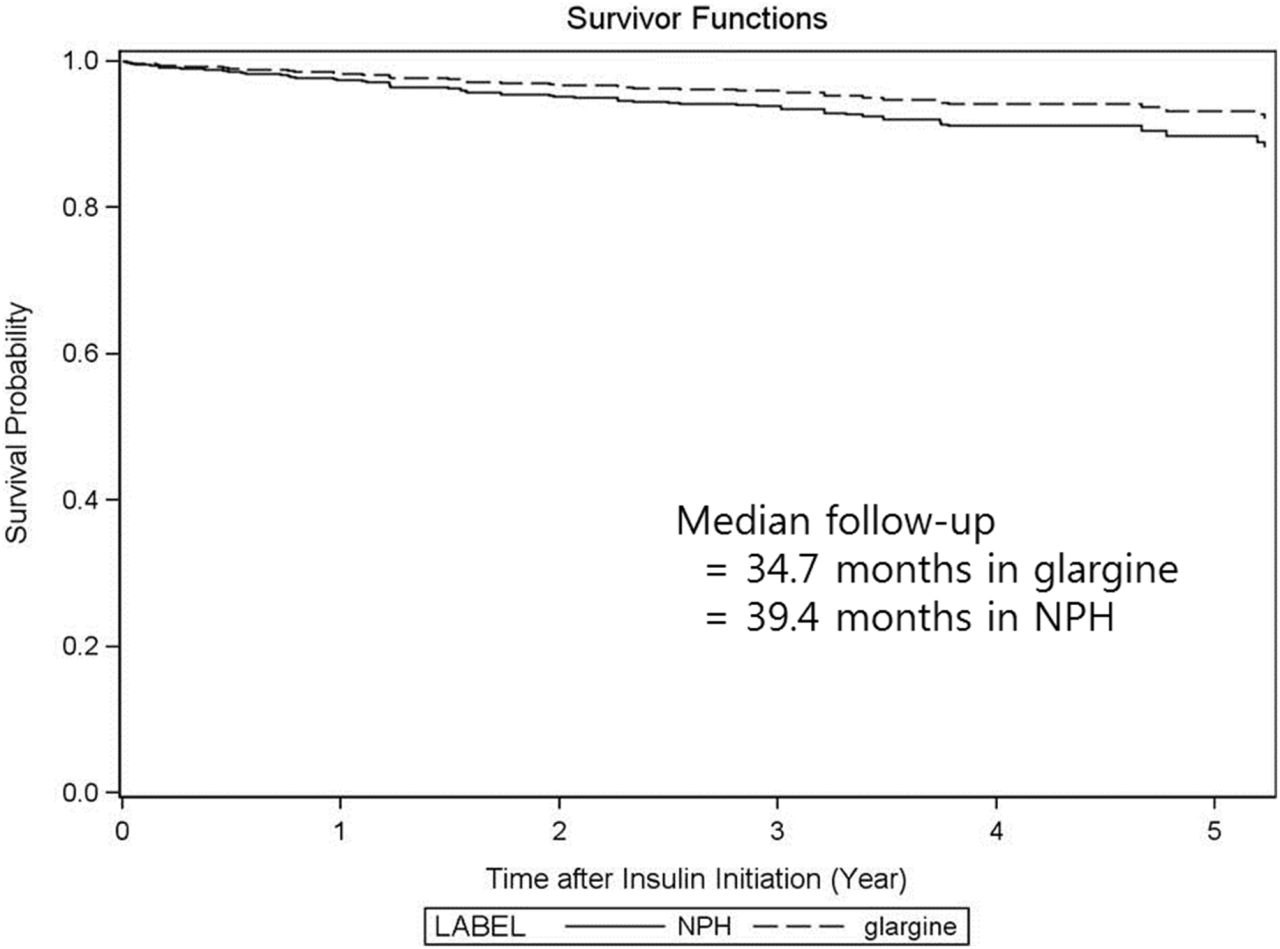
Cancer free survival curves in new glargine and NPH insulin users.

There is a possibility that diagnosis of cancer during the first year of follow-up may not be associated with insulin treatment. Among 56 patients who developed cancer during follow-up period, 8 in the glargine group and 8 in the NPH group developed cancer within the first year of treatment. There was no significant difference in risk of cancer incidence between the insulin glargine and NPH use excluding these cases. The cancer free survival curves between new users of insulin glargine and new users of NPH is shown in **Figure 1**.

During follow-up, 5.4% were switched to other long-acting insulin, and 12.5% stopped taking long-acting insulin before the study ended. Cumulative insulin dose was calculated until these points. The data with cumulative insulin dose was analyzed in **Table 3** (As-Treated Analysis). Since this needed to be performed as an as-treated analysis, the study subjects (n = 772) in **Table 3** is slightly fewer than that (n = 882) in **Table 2**, where everybody is included. The numbers of subjects in **Table 3** reflect the number of participants in each cumulative dose category: i.e., every patient is counted in the first dose category (n = 503 in glargine and n = 269 in NPH group) because everyone in this analysis had some exposure to drug. If these patients experience a cancer event before they reached a cumulative dose of 10 K, then their cancer event is counted in this dose category as well. The numbers for the second dose category (n = 391 in glargine and n = 210 in NPH group) reflect the number of patients who had a cumulative dose of at least 10 K; these 601 patients are a subset of the 772 patients who are in the first (0–10 K) dose category. In the Cox proportional hazards models adjusted for age and gender, no association of increased cancer risk with increased insulin dose was found (**Table 3**).

**Table 3:** Association between long-acting insulin use and cancer for new users by dose category (As-Treated Analysis).

Dose Category	Treatment	N	CancerEvents	TotalPerson-years	Incidence rateper 100person-years	Adjusted HRand 95% CI
≤10 K	Glargine	503	12	607.4	1.98	1.20 (0.38, 3.75)
	NPH	269	4	248.1	1.61	
10 K−<20 K	Glargine	391	3	282.6	1.06	0.71 (0.15, 3.43)
	NPH	210	4	142.7	2.80	
20 K−<50 K	Glargine	265	5	273.4	1.83	0.86 (0.25, 3.03)
	NPH	165	5	194.8	2.57	
≥50 K	Glargine	97	0	141.6	0.00	–
	NPH	86	4	150.1	2.66	

Hazard ratios and their 95% CIs were obtained from Cox proportional hazards models adjusted for gender and age at the start of the dose category.

CI, confidence interval; HR, hazard ratio; IR, incidence rate per 100 person-years.

Patients were followed until the first occurrence of one of the following events: the patient switched treatments or augmented with another long-acting insulin, the patient developed cancer, death, the study ended, or one year after the patient’s last visit in the database.

Only patients with dose data for the insulin treatment that they initiated are included in this analysis.

## Discussion

In this study of new insulin users (average age, 60 years; average duration of diabetes, 8 years), treatment with glargine insulin did not increase incidence of cancers when compared with NPH users after adjusting for baseline cancer risk factors including BMI and smoking status. Moreover, further adjustment of insulin dose did not affect this neutral effect of glargine insulin on incidence of cancers.

Since the four studies published in journal *Diabetologia* in 2009 created such a concern for cancer risk with insulin glargine use [1]–[4], several new studies have been published in regard to this issue, which are still inconclusive and have their own limitations [5], [7], [8], [10]–[12]. In a retrospective cohort study of patients with type 2 diabetes registered with the US Medicare, the data on important clinical risk factors for cancer including smoking status and obesity degree were not adjusted for [10]. In the study using the French National Healthcare Insurance system database, smoking status was not accounted for [8]. Another study using pharmacy dispensing data in the Netherlands had a likelihood of allocation bias and lower adherence to insulin glargine [7]. More recently, two large cohort studies from the French health insurance information system and the Inovalon Medical Outcomes Research for Effectiveness and Economics Registry showed no increased risk of any cancer in insulin glargine users compared with other long-acting insulin users [14], [15].

In contrast to these previous studies, we retrieved an array of relevant clinical and biochemical information such as baseline BMI, smoking status, concomitant medications, and cancer screening from the EMR, and performed the final regression model adjusted with this complete data. For smoking status in particular, we defined smoking status using the Natural Language Processing from the EMR discharge summaries [18]. It is important to point out that none of these variables were observed to confound the association between insulin use and cancer incidence.

For this study, we started with more than 20,000 patients and after careful selection process we only had 54 cancers cases. This suggests that with careful selection of patients, only a very small proportion are valid for looking at specific interactions like the one between glargine dose and cancer incidence. This also indicates importance of meticulous selection and adjustments to attain decisive answer to this unclear interaction.

More recently, the ORIGIN study showed that there was no increase of cancer incidence with glargine treatment over 6 years in subjects with prediabetes or early diabetes when compared to standard-care [13]. But the ORIGIN study population consisted of people who would not normally be prescribed insulin and the study had no active comparator such as NPH insulin. This may explain the significant differences in the baseline characteristics between ORIGIN and our study including comorbidities, diabetic complications, and other medications [5]. Indeed, there is the possibility that physicians tend to start or switch to insulin glargine in patients who are already more prone to developing cancers [6]. Patients who are generally less healthy are more likely to be prescribed easily administered daily insulin glargine than other types of insulin; this allocation bias is one of the limitations of previous studies [1], [6], [20], [21].

Our work yielded contrasting results using an active comparator study design. That is, the study mimics a treatment decision between two long acting insulins rather than comparing treated with untreated patients. Even hat physicians do not channel specific patients based on their BMI, smoking status, nor HbA1c preferentially to any of the long acting insulins compared here.

Several studies have shown a correlation between glargine dose and cancer risk [1], [22]. In order to investigate this relationship, we prospectively categorized cumulative insulin dose and found that adjustment of insulin dose did not change the neutral effect of glargine insulin on the incidence of cancer.

In this context, we had several advantages in the study design. We utilized new NPH users as an active comparator and there was no significant difference between groups with regard to diabetes severity and other potential cancer risks. We also adopted sophisticated methodology to perform a retrospective longitudinal study using EMR data. We applied strict and innovative criteria to obtain new users of glargine and NPH without prevalent cancer during 19 months prior to insulin initiation. This latent period helped us exclude indolent cancer cases in which cancer might be already present before diagnosis. Using this process, new glargine or NPH users without indolent cancers could be selected precisely, which suggests that these methods can be used as an example for future studies attempting to utilize EMR data. We also accounted for clinical visits and hospitalizations, which might have led to higher chance of cancer detection. We believe that the method used in this study can act as a potential model for others who may try to use EMR data for pharmacoepidemiologic study.

There are also several limitations of this study. First, the follow-up duration was not long enough to estimate the risk of some cancers although our average length of follow-up is comparable to previous studies [22]. Moreover, the number of study subjects was no as substantial as that of recent studies [14], [15]. In addition, due to the limited number of cancer cases, we could not evaluate individual cancer risk. However, from a clinical aspect, arguably patients do not really care what cancer they develop among those ascertained. Second, there is a possibility that some patients can be treated in other hospitals. But the recruitment strategy of selecting loyal patients is likely to reduce this chance. Fourth, the compliance of insulin medication was not examined.

In conclusion, no cancer signal with insulin glargine was found in this carefully characterized clinical cohort with diabetes. While our study is limited in size, we avoided potential for major distortions (or bias) by implementing a new user, active comparator study design. Our study thus adds to the evidence that insulin glargine does not increase the risk for any cancer outcomes when compared with its main treatment alternative, NPH insulin.

## Supporting Information

File S1 **A supporting figure (Figure A) and table (Table A).** Figure A, Study subject selection process using Eligibility Cohort Algorithm. Table A, Frequency of ICD-9 codes that define “Any Cancer”.
(RTF)

## References

[1] Hemkens LG, Grouven U, Bender R, Gunster C, Gutschmidt S, et al (2009) Risk of malignancies in patients with diabetes treated with human insulin or insulin analogues: a cohort study. Diabetologia 52: 1732–44. 19565214 10.1007/s00125-009-1418-4 PMC2723679

[2] Jonasson JM, Ljung R, Talback M, Haglund B, Gudbjornsdottir S, et al (2009) Insulin glargine use and short-term incidence of malignancies-a population-based follow-up study in Sweden. Diabetologia 52: 1745–54. 19588120 10.1007/s00125-009-1444-2

[3] Colhoun HM (2009) Use of insulin glargine and cancer incidence in Scotland: a study from the Scottish Diabetes Research Network Epidemiology Group. Diabetologia 52: 1755–65. 19603149 10.1007/s00125-009-1453-1 PMC2723678

[4] Currie CJ, Poole CD, Gale EA (2009) The influence of glucose-lowering therapies on cancer risk in type 2 diabetes. Diabetologia 52: 1766–77. 19572116 10.1007/s00125-009-1440-6

[5] Chang CH, Toh S, Lin JW, Chen ST, Kuo CW, et al (2011) Cancer risk associated with insulin glargine among adult type 2 diabetes patients–a nationwide cohort study. PLoS One 6: e21368. 21738645 10.1371/journal.pone.0021368 PMC3124499

[6] Pocock SJ, Smeeth L (2009) Insulin glargine and malignancy: an unwarranted alarm. Lancet 374: 511–3. 19616841 10.1016/S0140-6736(09)61307-6

[7] Ruiter R, Visser LE, van Herk-Sukel MP, Coebergh JW, Haak HR, et al (2012) Risk of cancer in patients on insulin glargine and other insulin analogues in comparison with those on human insulin: results from a large population-based follow-up study. Diabetologia 55: 51–62. 21956710 10.1007/s00125-011-2312-4 PMC3228952

[8] Blin P, Lassalle R, Dureau-Pournin C, Ambrosino B, Bernard MA, et al (2012) Insulin glargine and risk of cancer: a cohort study in the French National Healthcare Insurance Database. Diabetologia 55: 644–53. 22222504 10.1007/s00125-011-2429-5 PMC3268990

[9] Rosenstock J, Fonseca V, McGill JB, Riddle M, Halle JP, et al (2009) Similar risk of malignancy with insulin glargine and neutral protamine Hagedorn (NPH) insulin in patients with type 2 diabetes: findings from a 5 year randomised, open-label study. Diabetologia 52: 1971–3. 19609501 10.1007/s00125-009-1452-2 PMC2723677

[10] Morden NE, Liu SK, Smith J, Mackenzie TA, Skinner J, et al (2011) Further exploration of the relationship between insulin glargine and incident cancer: a retrospective cohort study of older Medicare patients. Diabetes Care 34: 1965–71. 21775752 10.2337/dc11-0699 PMC3161263

[11] Buchs AE, Silverman BG (2011) Incidence of malignancies in patients with diabetes mellitus and correlation with treatment modalities in a large Israeli health maintenance organization: a historical cohort study. Metabolism 60: 1379–85. 21696791 10.1016/j.metabol.2011.05.002

[12] Suissa S, Azoulay L, Dell'Aniello S, Evans M, Vora J, et al (2011) Long-term effects of insulin glargine on the risk of breast cancer. Diabetologia 54: 2254–62. 21614572 10.1007/s00125-011-2190-9

[13] Basal Insulin and Cardiovascular and Other Outcomes in Dysglycemia. N Engl J Med 367: 319–28. 22686416 10.1056/NEJMoa1203858

[14] Fagot JP, Blotiere PO, Ricordeau P, Weill A, Alla F, et al (2013) Does insulin glargine increase the risk of cancer compared with other basal insulins?: A French nationwide cohort study based on national administrative databases. Diabetes Care 36: 294–301. 22966091 10.2337/dc12-0506 PMC3554310

[15] Sturmer T, Marquis MA, Zhou H, Meigs JB, Lim S, et al (2013) Cancer incidence among those initiating insulin therapy with glargine versus human NPH insulin. Diabetes Care 36: 3517–25. 23877991 10.2337/dc13-0263 PMC3816915

[16] Atlas SJ, Chang Y, Lasko TA, Chueh HC, Grant RW, et al (2006) Is this "my" patient? Development and validation of a predictive model to link patients to primary care providers. J Gen Intern Med 21: 973–8. 16918744 10.1111/j.1525-1497.2006.00509.x PMC1831616

[17] Setoguchi S, Solomon DH, Glynn RJ, Cook EF, Levin R, et al (2007) Agreement of diagnosis and its date for hematologic malignancies and solid tumors between medicare claims and cancer registry data. Cancer Causes Control 18: 561–9. 17447148 10.1007/s10552-007-0131-1

[18] Regan S, Meigs JB, Grinspoon SK, Triant VA (2012) Determinants of Smoking and Quitting in an HIV Cohort Using a Validated Natural Language Processing Tool. Cardiovascular Disease Epidemiology and Prevention|Nutrition, Physical Activity and Metabolism (Abstract).

[19] Sturmer T, Joshi M, Glynn RJ, Avorn J, Rothman KJ, et al (2006) A review of the application of propensity score methods yielded increasing use, advantages in specific settings, but not substantially different estimates compared with conventional multivariable methods. J Clin Epidemiol 59: 437–47. 16632131 10.1016/j.jclinepi.2005.07.004 PMC1448214

[20] Gough SC, Belda-Iniesta C, Poole C, Weber M, Russell-Jones D, et al (2011) Insulin therapy in diabetes and cancer risk: current understanding and implications for future study: proceedings from a meeting of a European Insulin Safety Consensus Panel, convened and sponsored by Novo Nordisk, held Tuesday October 5, 2010 at The Radisson Edwardian Heathrow Hotel, Hayes, Middlesex, UK. Adv Ther 28 Suppl 5 1–18. 10.1007/s12325-011-0047-8 21863297

[21] Bloomgarden ZT (2011) Insulin concerns and promises. Diabetes Care 34: e100–e106. 21617099 10.2337/dc11-0591 PMC3114335

[22] Mannucci E, Monami M, Balzi D, Cresci B, Pala L, et al (2010) Doses of insulin and its analogues and cancer occurrence in insulin-treated type 2 diabetic patients. Diabetes Care 33: 1997–2003. 20551014 10.2337/dc10-0476 PMC2928350

