# Supplementary material for: Electronic Medical Record Cancer Incidence over Six Years Comparing New Users of Glargine with New Users of NPH Insulin
Source: PLoS One. 2014 Oct 20;9(10):e109433. doi: 10.1371/journal.pone.0109433 (PMC4203726; doi:10.1371/journal.pone.0109433)
Supplement: File S1 — A supporting figure (Figure A) and table (Table A). Figure A, Study subject selection process using Eligibility Cohort Algorithm. Table A, Frequency of ICD-9 codes that define “Any Cancer”. (RTF) [file pone.0109433.s001.rtf]

Figure A. Study subject selection process using Eligibility Cohort Algorithm


Table A. Frequency of ICD-9 codes that define “Any Cancer”.	
	ICD-9-CM diagnostic codes	Incident Cancer Codes in MGH data	Number of Patients	
Breast	174.X, 233.0	174.9	5	
Prostate	185.X	185	5	
Colon	153.X	153.4
153.8
153.9	1
2
1	
Pancreas	157	157
157.2
157.9	1
1
2	
Lung, trachea and bronchus	162	162.9	4	
Bladder	188	188.8
188.9	2
1	
Kidney	189	189	4	
Lymphoid and histiocytic tissue	202	202.78
202.8
202.84
202.88	1
1
1
1	
Multiple myeloma	203	203
203.01	2
1	
Other Cancers	Any code from the following list except those reported above: 140.0–208.92 ( except 173.X), 209.00 – 209.36, 209.70-209.79, 233.0, 236.0, 237.0-237.1, 237.5-237.6, 237.72, 237.9, 238.4, 238.6, 238.7X (all but 238.78), 239.6, 239.7, 273.2, 273.3, 277.89, 288.4, 795.06, 795.16, 796.76	141.5
146.9
154.1
155
159
172.5
179
182
193
197.2
198.5
199.1
208.9
231
238.4
238.75
238.79
239.6	1
1
1
1
1
1
1
1
1
1
1
1
1
1
1
1
1
1	
Total			54	
Incident cancers were identified using the Setoguchi algorithm which requires two cancer codes within two months.  The second incident cancer code is reported in this summary.

Codes are based on the National Cancer Institute's Surveillance Epidemiology and End Results (SEER) 2011 Case finding List (http://seer.cancer.gov/tools/casefinding) with the exclusion of codes for a personal history of cancer, benign neoplasms and carcinoma in situ except breast carcinoma in situ. 	
